# The effect of *Valeriana officinalis* tea on sympathovagal tone and cardiac function in healthy volunteers: A semi-experimental study

**DOI:** 10.22038/AJP.2024.24974

**Published:** 2025

**Authors:** Seyed Mehran Hosseini, Shahab Zanganeh, Marzieh Qaraaty

**Affiliations:** 1 *Neuroscience Research Center, Department of Physiology, School of Medicine, Golestan University of Medical Sciences, Gorgan, Iran *; 2 *Student Research Committee, Golestan University of Medical Sciences, Gorgan, Iran*; 3 *Department of Traditional Medicine, School of Traditional Medicine, Shahid Beheshti University of Medical Sciences* *, * *Tehran* *,* * Iran *; 4 *Clinical Research Development Center (CRDC), Sayad Shirazi Hospital, Department of Persian Medicine, School of Medicine, Golestan University of Medical Sciences, Gorgan, Iran *

**Keywords:** Valeriana officinalis tea, Heart rate, Blood pressure, Sympathovagal, Electrocardiogram

## Abstract

**Objective:**

The use of herbal teas can affect some physiological parameters of the body. Valerian has been used as a valuable medicinal plant. There are reports about sedative and sleep-inducing effects of *Valeriana officinalis *L. (VOT) on the nervous system. But in relation to its possible effect on the autonomic nervous system, the available information is limited. This study aimed to determine the effect of VOT on sympathovagal tone based on heart rate variability indices.

**Materials and Methods:**

In this semi-experimental study, 12 healthy volunteers were enrolled. At first, the participants received 50 ml of water as the control group, and then after the clearance time, they received VOT with a dilution of 50% with the same temperature and volume and were considered the intervention group. Assessment of sympathovagal tone was performed in terms of heart rate variability indices. There were 5 recording steps: baseline, after drinking water, and 5, 20, and 30 minutes after drinking VOT.

**Results:**

The mean±SD of the average heart rate per minute at the five recording steps after VOT was 65.4±15.5, 63.5 ±14.6, 62.7±15.6, 61.8±16.09, and 60.9 15.2, respectively (p<0.05). The average arterial systolic pressure at the five recording steps after VOT was 119.4±7.4, 117.9± 9, 114.3±7.9, 113.8±8.6, and 114±6.5 mmHg, respectively.

**Conclusion:**

A single cup of VOT significantly decreased the heart rate. This effect may be associated with a decrease in sympathetic activity and an increase in parasympathetic activity.

## Introduction

The use of herbal teas can affect some physiological parameters of the body. Blood pressure, plasma glucose, gastrointestinal motility and/or secretion, and many other physical or chemical physiological parameters may be changed following drinking herbal teas. The activity of the autonomic nervous system (ANS) may also be challenged by medicinal plants. The sympathovagal balance can be monitored by invasive and non-invasive methods. However, the differential assessment of alternation in ANS activity needs separate consideration of sympathetic and parasympathetic divisions. Heart rate variability (HRV) is a manifestation of sympathovagal tone (Tiwari et al., 2021). HRV measures the fluctuation of the intervals between consecutive heart beats. The main part of HRV is related to the influences of the ANS on the sinus node and the minor parts of HRV are related to cellular, hormonal, and thermoregulatory oscillations. HRV analysis can provide many different indices some of which being specific markers for only one division of ANS (Shaffer and Ginsberg, 2017). Instantaneous speed of the heart is used for definition and calculation of time and frequency domain indices of HRV. Some of the time domain HRV indices are Standard deviation of all normal RR intervals recorded in a time interval, expressed in ms the standard deviation of normal sinus beat-to-beat intervals (SDNN), the root-mean square of differences between adjacent normal RR intervals in a time interval, expressed in ms (rMSSD), and the percentage of adjacent RR intervals with a difference of duration greater than 50 ms (pNN50). Some of the HRV indices in the frequency domain are high frequency (HF), low frequency (LF), and LF/HF ratio. HF ranges from 0.15 to 0.4 Hz. and corresponds to the respiratory modulation and is an indicator of the performance of the parasympathetic tone on the heart. LF ranges between 0.04 and 0.15 Hz. and is due to the overall balance of the parasympathetic and sympathetic components on the heart, with a predominance of the sympathetic ones. The LF/HF ratio reflects the absolute and relative changes between the sympathetic and parasympathetic components of the ANS, by characterizing the sympathetic-vagal balance on the heart (Malik et al., 1996).

*Valeriana officinalis* L. (VO) is one of the medicinal plants whose dried root is used in the form of tablet or tea to induce sleep (Francis and Dempster, 2002; Bent et al., 2006; Leach and Page, 2015), reduce anxiety symptoms (Rezaie et al., 2010; Borrás et al., 2021; Andreatini et al., 2002). No complications, unwanted effects, or sensitivity have been reported in the short term clinical studies conducted with the extract of this plant (Chandra Shekhar et al., 2024; Azizi H et al., 2020). It is considered safe even during pregnancy and breastfeeding. In Australia, it is known as part of group A, and the Food and Drug Administration (FDA) has declared its entry into food without hindrance (Patočka and Jakl, 2010; Mozaffarian, 1997; Hendriks et al., 1981; Ernst, 2000). The active ingredients present in VO root and rhizome include alkaloids valerine, valerianin, valerate and isovaltrate and volatile oils alpha and beta pinene, camphene, bonole, eugenol, isoeugenol, bisabolene, caryophyan, valeral, valerianol and valeric, and chlorogenic and caffeic acids (Murphy et al., 2010; Shinjyo et al., 2020). The sedative effects of VO are related to the volatile oils of valeranal and valeric acid and valpotriate compounds (Ziegler et al., 2002). Valerian acid has inhibitory effect on gamma-aminobutyric acid (GABA) aminotransferase enzyme and increases the concentration of GABA in brain tissue and it also is known as an agonist of GABA receptors (Patočka and Jakl, 2010; Barnes et al., 2007; Mozaffarian, 1997). In an animal model, intravenous administration of VO extract reduced the arterial blood pressure through a vasodilatory mechanism by reducing peripheral vascular resistance. This effect was independent of the endothelium and was mediated through the direct relaxing effect of vascular smooth muscles and it did not cause significant changes in heart rate (Gilani et al., 2005). At the cellular level, VO inhibits the cell depolarization currents and causes potassium channels to open and calcium channels to block (Mirabi et al., 2011). 

There are few reports about VO effect on heart rate and the results are different (Chen et al., 2015; Sedighi et al., 2023). At the time of the present study, all articles in the literature reported the effect of VO only on average heart rate. The results of these articles included no change or some changes in mean heart rate. If the mean heart rate does not change, monitoring the instantaneous heart rate and analyzing its changes can provide indicators for quantitative assessment of changes in the sympathetic and vagal tone of the heart. The effect of VO on HRV and sympathovagal tone has not been reported yet. The anti-anxiety and sedative effect of VO is dose-dependent, and drinking one cup of VOT that is equal to 50 ml of 2.5% VOT may only cause mild effects. There is little information about the effect of this plant on the autonomic nervous system. Therefore, this study was conducted with the aim of determining the effect of drinking 50 ml of 2.5% VOT on the autonomic system and sympathovagal tone based on HRV indices. 

## Materials and Methods

### Ethical considerations

Ethical Committee of Golestan university of medical sciences approved this study with the code of IR.GOUMS.REC.1397.103 (approval date: 2018-07-29) and registered in Iranian Registry for Clinical Trials IRCT20110907007511N6. All participants signed the written informed consent to participate in the trial.

### Plant material

The samples of cultivated VO were purchased from a local medicinal plants market, Tehran, Iran. The sample was identified and deposited under the herbarium number PMP-6296, by a botanist at the Plant Biosystematics Department of Golestan University, Gorgan, Iran.

### High performance liquid chromatography (HPLC) analysis of VOT

A C18 reversed-phase HPLC analysis (Agilent 1100, UV-FLD, United States) was performed on quercetin as a marker of secondary metabolites in VOT. The gradient elution with A and B solvents (A: acetonitrile 10%, and water; B: acetonitrile 55%, and water) at a flow rate of 1 ml/min was used as the mobile phase. The column temperature and UV/V detector were set at 30 centigrade and 270 nm, respectively.

### Preparation of VOT

VOT was prepared by adding two tablespoons (5 g) of the powdered root of the plant to 100 ml of boiling drinking water. Following 20 min of brewing, and a 20-min thermal equilibrium time, it was administered in the form of tea with a dilution of 50% and a volume of 50 ml for each participant. In a pilot group, the possible effect of the bitterness of VOT on HRV was tested. For this, VOT was tasted and thrown out (not swallowed) by the participant. The HRV indices were recorded and compared with the baseline values in the time intervals of one, five, and fifteen minutes. In addition, the effect of the act of swallowing on HRV indices was also investigated separately in a pilot form.

### Study design

**Figure 1:**
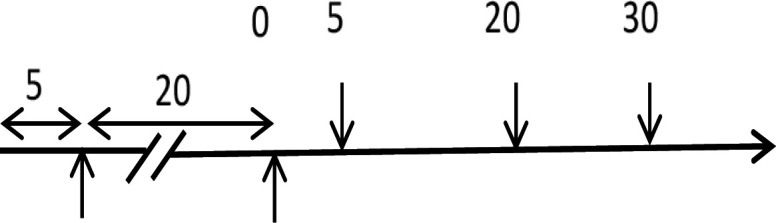
The scheduling of study and data collection. The numbers are data collection times (in minutes). The recording started before the water drinking, then after it, and then at 5, 20, and 30 min after VOT drinking.

This semi-experimental and descriptive-analytical study was conducted on 12 healthy male volunteers who were medical students. Due to follicular and/or luteal phase hormonal changes in women, which may have a potential influence on HRV indices, only male volunteers were recruited in this study. All participants avoided tea, coffee, or caffeine in the last 24 hr prior to data collection. Tests were performed between 9 and 10 Am for all participants. This study was performed in the Physiology Laboratory of the Faculty of Medicine of Golestan University of Medical Sciences in 2021. The scheduling of study design and data collection is shown in Figure 1.

The sample size was determined based on the formula n= (Z_1-α/2_)^2^σ^2^/d^2^ using the G power software by the following inputs: one tile, effect size 1, α error probability 0.05, and power (1-β error probability) 0.95 (Shahinfar et al., 2016). The inclusion criteria consisted of having no drug history, no smoking, and negative history of the disease, or hospitalization in the last few months. The exclusion criteria included having more than 10% artefact in Electrocardiogram (ECG) traces and the existence of any abnormal rhythm in ECG. 

Although there were medicinal forms of VO root extract such as Sedamine tablets in pharmacy; to be more similar to the consumption of this plant as an herbal tea, it was used in this form.

When a volunteer was fully settled on the bed, the ECG electrodes, the respiratory belt transducer, and the blood pressure cuff were connected to them. Power Lab 4/26 and Dual Bio/Stim ML 408 (AD instrument, Australia) were used to record ECG and for respiration monitoring. The settings for recording the ECG were as follows: the sampling rate was 1 kHz and the minimum duration of the recorded signals to calculate the indicators of HRV was 5 min. To adjust the effect of the total power inequality on the absolute values of the low and the high-frequency component of the spectrum, these values were adjusted based on the amount of very low frequencies according to the following equation: (LF or HF [ms2]/ [total power [ms^2^]-VLF [ms^2^]). 

We used a digital filter to remove fluctuations and artifacts. Analysis of HRV was done offline with the Lab chart 7. Blood pressure was recorded from the right brachial artery by an oscillometric sphygmomanometer (Omron M6 comfort, Japan) with an accuracy of ± 3 mmHg.

### Statistical analysis

The data are presented as mean ± SD and statistical significance was considered at p<0.05. The parametric repeated measures ANOVA or the non-parametric Friedmann test was used for statistical comparisons made by SPSS version 20.

## Results

### HPLC analysis

**Figure 2:**
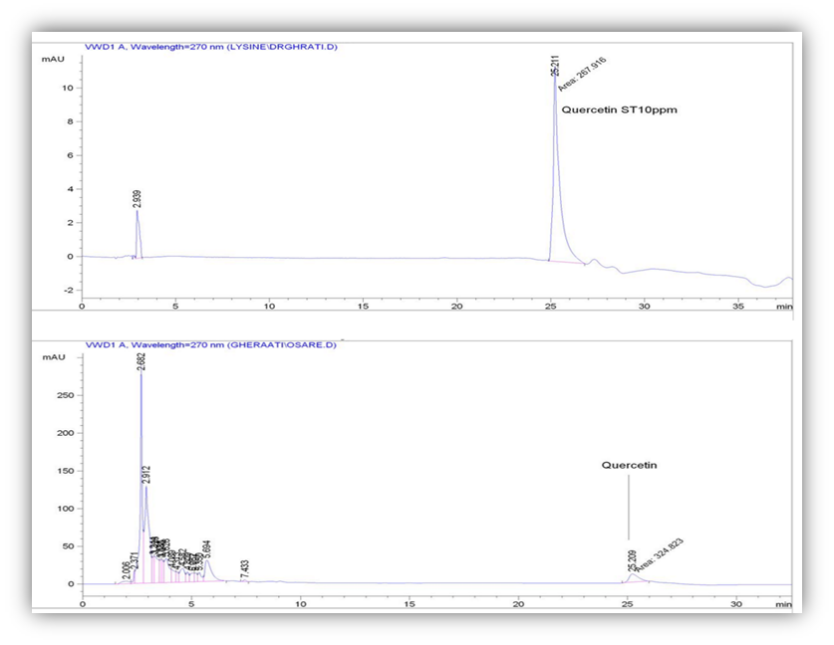
Reversed-phase HPLC-PDA analyses of the VOT and Quercetin; chromatographic profiles are reported at 270 nm.

The HPLC chromatogram showed the quercetin with a retention time of 25.21 min as an ingredient of VOT (Figure 2). Also, the quercetin amount in the VOT was 0.73 μg/g (Figure 2).

### Biological analysis

**Figure 3:**
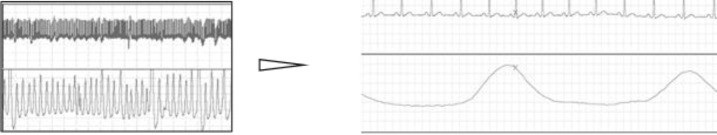
A recorded trace of ECG (top row) and respiratory movements (bottom row) in compressed (A) and uncompressed (B) time interval format.

The mean±SD values of age, weight and height of the volunteers were 24.5±2.03 years, 73.2±8.2 kilograms, and 176.2±4.3 centimeters, respectively. A recorded trace of ECG and respiratory movements in compressed and uncompressed time interval format is shown in Figure 3 and Table 1.

The mean±SD of the respiratory rate and the amplitude of chest wall movement during inspiration and expiration in the five experimental conditions were 16.8±0.63, 16.9±0.57, 17.2±0.63, 17.2±0.63, 17.1±0.74 per minute, and 65.2±15, 64±17.7, 62±16, 59±14, and 60±16 millivolts, respectively. The mean±SD of the time-domain and normalized frequency-domain indices of HRV are shown in Table 2 and the changes in systolic blood pressure (SBP) in five experimental conditions are shown in Figure 3.

Only two variables of HR and NN50 showed a significant difference compared among the five experimental conditions. (with 95% confidence interval). Compared to the water, VOT decreased the HR; this decrement seemed to be time-dependent and was more prominent 30 min after drinking VOT compared to 20 and 5 min, respectively. The results of the analysis of different variables of the study according to the type of statistical test are shown in Table 3.

## Discussion

In the current study, VOT exerted modulatory effects on sympathovagal tone as a natural agent. Our data indicated that the sympathovagal balancing by VOT was regulated via a reduction of HR, amplification of the parasympathetic tone, suppression of sympathetic activity and reduction of the SBP.

Owing to the multiple pharmacological effects of VO ingredients such as valepotriates, they have aroused much interest in the treatment of nervous system and cardiovascular disorders through GABA receptors, and vasodilatory effect on vascular smooth muscle (Gilani et al., 2005; Mozaffarian, 1997; Wang et al., 2017). Twe recent study revealed that VO contained valepotriates and exerted cardioprotective effects such as reducing BP and HR (Gilani et al., 2005; Chen et al., 2015). Consistent with the present research, the sympathovagal balancing properties and cardioprotection of VO may be related to its active constituents including valepotriates and amino acids. In addition, other compounds might be involved in the biological effects of VO (Wang et al., 2017; Chen et al., 2015). The findings of the current study showed that even a single dose of VO in the form of herbal tea (not in the form of concentrated or processed ingredients drug) can be effective on sympathovagal tone. However, VO maintained the sympathetic-parasympathetic balance at 20 and 30 minutes as shown in the SDNN and RMSSD indexes. Previous studies indicated that SDNN reduction reflects the diminishing sympathetic tone, which may be related to the left ventricle function by directly decreasing BP (Shim et al., 2014; Bolívar, 2013). Additionally, parasympathetic modulation of the heart during sympathetic tone reduction is thought to be mediated by RMSSD reduction and the sympathovagal balance (Shim et al., 2014). 

**Table 1:** The mean±SD of the ECG parameters in the experimental conditions baseline, after water drinking, and after the VOT drinking at 5, 20, and 30 minutes (n=12).

**Experimental**	**Herat rate (bpm)**	**SBP (mmHg)**	**DBP (mmHg)**	**HF (Hz)**	**LF (Hz)**	**Total Power (ms** ^2^ **)**
Baseline	66 (15)	119 (7)	70 (7)	3481 (2604)	2062 (1646)	9222 (6477)
Water	64 (15)	118 (9)	72 (9)	4569 (4839)	2675 (2481)	12882 (11480)
Valerian 5 min	63 (16)	114 (7)	71 (10)	1802 (1647)	1769 (1114)	6698 (4363)
Valerian 20 m	62 (16)	113 (8)	74 (9)	5591 (4641)	2441 (1588)	11112 (8856)
Valerian 30 m	61 (15)	114 (6)	70 (8)	3040 (2409)	2619 (2180)	9824 (4860)

bpm: beat per minute

**Table 2:** The mean±SD of the time-domain and normalized frequency-domain indices of the HRV spectrum in five experimental conditions (n=12)

**Experimental**	**HFnu**	**LFnu**	**LFHFr**	**SDNN**	**SDdeltaNN**	**SDNNSDdeltaNNr**	**RMSSD**	**NN50**
Baseline	44.6(12)	36(17)	0.905(0.77)	88.7(30)	105(51)	0.972(0.36)	105(51)	971(260)
Water	46(10)	34(16)	0.807(0.55)	108(44)	130(72)	0.950(0.36)	130(72)	995(256)
Valerian 5	41(10)	43(20)	1.204(0.781)	85(26)	96.2(45)	0.957(0.22)	96(45)	1019(286)
Valerian 20	48(14)	36(16)	0.850(0.6)	92.5(38)	116(60)	0.890(0.27)	116(60)	1039(301)
Valerian 30	47(12)	37(15)	0.860(0.5)	99.4(26)	106(39)	1.013(0.41)	106(39)	1049(297)

NN: normal sinus beat-to-beat intervals, NN50: the number of pairs of successive NN intervals that differ by more than 50 ms, SDNN: the standard deviation of NN intervals, SD Delta NN: standard deviation of the differences between adjacent NN intervals, RMSSD: the root mean square of successive differences between adjacent NNs, SDNNSDdeltaNNr: standard deviation of successive differences between adjacent NNs, HFnu: normalized high frequency, LFnu: normalized low frequency, LFHFr: LF to HF ratio.

**Table 3:** Results of analysis of different variables in the study.

**Variable**	**p-Value**	**Visual**	**p-Value**	**p-Value**
	**Normal**	**Outlier**	**RM**	**Friedman**
TP*	0.04	No	-	0.27
HF*	0.00	No	-	0.15
HFnu**	0.12	No	0.25	-
LF*	0.00	No	-	0.49
LFnu*	0.02	No	-	0.40
LFHFr*	0.00	No	-	0.67
SDNN**	0.30	No	0.49	-
SDdeltaNN**	0.09	No	0.59	-
SDNNSDΔNNr*	0.00	No	-	0.30
RMSSD**	0.09	No	0.59	-
MaxNN**	0.08	No	0.68	-
MinNN**	0.09	No	0.18	-
RangeNN*	0.02	No	-	0.30
MeanNN*	0.00	No	-	0.06
NN50*	0.00	No	-	0.04***
HR*	0.04	No	-	0.04***
Normal beats*	0.02	No	-	0.20
Ectopic beats*	0.01	No	-	0.49
SBP**	0.43	No	0.93	-
DBP*	0.02	No	-	0.96
Respiratory Rate**	0.95	No	0.92	-
Respiratory amplitude*	0.19	No	-	0. 2

*non-normal distribution, **normal distribution, RM: repeated measure, ANOVA, ***p<0.05 as compared among the five experimental conditions

TP: total power; HF: high frequencies; HFnu: normalized high frequency; LF: low frequencies; LFnu: normalized low frequency, LFHFr: LF to HF ratio; SDNN: the standard deviation of NN intervals; SDDeltaNN: standard deviation of the differences between adjacent NN intervals; SDNNSDdeltaNNr: standard deviation of successive differences between adjacent NNs; RMSSD: the root mean square of successive differences between adjacent NNs; MaxNN: Maximum RR intervals; MinNN: Minimum RR intervals; RangeNN: Range of RR intervals; MeanNN: Mean of RR intervals; NN50: the number of pairs of successive NN intervals that differ by more than 50 ms HR: herat rate; SBP: systolic blood pressure; DBP: diastolic blood pressure

In line with the other studies, we showed that VOT decreased the sympathetic tone and parasympathetic tone at the first 5 minute of drinking VOT against to an increase after water drinking, probably via impacting on cardiac mechanoreceptor, and modulating inotropy effect through reducing heart rate and SBP. Moreover, VOT simultaneously increased the sympathetic tone and parasympathetic tone at 20 and 30 minutes, which indicated the sympathovagal balance effect of VO. Therefore, the beneficial cardioprotective roles (SBP and heart rate reduction) of VO are mediated via the autonomic nervous system pathways. 

In the frequency domain, VOT decreased normalized high frequency (HFnu) and increased normalized low frequency (LFnu) at the first 5 minutes after its drinking, while it increased HFnu and decreased LFnu at 20 and 30 minutes after drinking VOT compared to water. However, the results suggest relatively stable and basic sympathetic tone and activation of parasympathetic tone time-dependently in the VOT, which are in line with decreasing HR and BP. These results indicate that VO may regulate ANS by its antagonistic effects on the heart and may balance the secretion of norepinephrine (reduction) and acetylcholine (increase) and may activated the adrenergic receptors in the heart and coronary arteries, and finally reduced the heart rate and blood pressure (Mozaffarian, 1997; Barnes et al., 2007). This result corresponds with the previous findings that sympathovagal tone is involved in regulating cardiac function and dysfunction (Berg and Jensen 2011; Gordan et al., 2015).

Taken together, this study showed that VOT could effectively change some HRV variables. These potentially beneficial properties including BP and HR reduction are due to a sympathetic-parasympathetic balance effect of the VOT in the healthy participations. To clarify the precise function of the ANS involved in the cardio-modulatory effect of VOT, further studies are necessary to confirm the cross-talk between the GABAergic modulatory effects of VOT and sympathovagal tone pathways. This study had some limitations; only one dose and concentration of VOT was tested, while VOT may have dose-dependent effects on HRV and the sample size was small. It is suggested to consider dose-dependent effects on HRV in future large studies.

The major limitation of this study was the inability to analyze the major components of the extract by HPLC, which could give important insights to researchers.

## Abbreviations

VOT*: Valeriana officinalis* tea; HRV: heart rate variability; ECG: Electrocardiogram; GABA: gamma-aminobutyric acid; FDA: Food and Drug Administration; HF: high frequencies; LF: low frequencies, TP: total power; BPM: beat per minute; SBP: systolic blood pressure; DBP: diastolic blood pressure; NN: normal sinus beat-to-beat intervals; NN50: the number of pairs of successive NN intervals that differ by more than 50 ms; SDNN: the standard deviation of NN intervals; SD Delta NN: standard deviation of the differences between adjacent NN intervals; RMSSD: the root mean square of successive differences between adjacent NNs; SDNNSDdeltaNNr: standard deviation of successive differences between adjacent NNs; HFnu: normalized high frequency, LFnu: normalized low frequency, LFHFr: LF to HF ratio.
